# The complete mitochondrial DNA of *Gymnocypris potanini* Herzensten and comparative mitogenomic analyses in *Gymnocypris* species

**DOI:** 10.1080/23802359.2016.1225525

**Published:** 2016-11-22

**Authors:** Taiming Yan, Jiaxiang Hu, Yueping Cai, Sen Xiong, Zhide He, Song Li, Xiongyan Wang, Deying Yang, Zhi He

**Affiliations:** College of Animal Science and Technology, Sichuan Agricultural University, Chengdu, China

**Keywords:** *Gymnocypris potanini* Herzensten, mitochondrial genome, phylogenetic analyses

## Abstract

In the study, the complete mitochondrial genome of *Gymnocypris potanini* Herzensten was sequenced and compared with other *Gymnocypris* species. The mitochondrial genome, consisting of 16,749 base pairs (bp), encoded 13 protein-coding genes, 2 ribosomal RNAs, 22 transfer RNAs, and a noncoding control region, similar as that found in other *Gymnocypris* species. These results can provide useful information for further studies on taxonomic status, molecular systematics, and stock evaluation.

*Gymnocypris potanini* Herzensten is categorized into the family Cyprinidae, order Cypriniformes, only distributed in the upper reaches of Mingjiang River and the Lancang River (Yue et al. 2000). It usually lives in some plateau river where the water temperature is very low even in the summer. In recent years, the studies on this species are only limited to few reports on the taxonomic characters and distribution(Yue et al. 2000), and a few basic biological data (Song et al. 2006; Wu et al. 2001). Therefore, some basic biological data including genetic information should be further studied, which may be useful in systematics, resource protection, and development.

In the present study, one specimen *G. potanini* Herzensten chosen for mitochondrial genome analysis were collected from the upstream of Mingjiang River (N: 31°43′57.82″, E: 103° 2′11.37″) (Specimen is stored in Aquaculture Department of Sichuan Agricultural University, number gph20150102). Primers were designed for polymerase chain reaction (PCR) amplification and sequencing on the basis of the mitogenome sequence of *G. eckloni* Herzensten (GenBank Accession No. JQ0042791) (Qi et al. 2013). The complete mt genome of *G. potanini* Herzensten was 16,749 bp and has been deposited in GenBank with Accession No. KX424966. The mitochondrial genome encoded 13 protein-coding genes, 2 ribosomal RNAs, 22 transfer RNAs, and a noncoding control region, as those found in other *Gymnocypris* species (Qi et al. 2013; Qiao et al. 2014; Zhang et al. 2016). The nucleotide composition of the genome of *G. potanini* Herzensten is 28.5% for A, 27.2% for T, 18.2% for G, and 26.1% for C, with a low A + T content of 55.7%. Except for the *nad6* and eight tRNA genes *(tRNA-Gln*, *tRNA-Aln*, *tRNA-Asn*, *tRNA-Lys*, *tRNA-Tyr*, *tRNA-SerUCN*, *tRNA-Glu*, and *tRNA-Pro*) encoded on the light-strand, all others genes were encoded on the heavy-strand. This is a typical gene arrangement conforming to the other *Gymnocypris* species and vertebrate consensus (Boore 1999; Qi et al. 2013; Qiao et al. 2014).

All genes use ATG as start codon, except *cox1* use GTG, which is also discovered in other *Gymnocypris* species (Qi et al. 2013; Qiao et al. 2014). Most open reading frames ended with two types of complete stop codons TAA and TAG, whereas few genes (including *cox2*, *nd4* and *cob*) had an incomplete stop codon T––. These results showed that the PCGs were stable among the *Gymnocypris* species.

Based on the combined nucleotide sequence data of 12 heavy-strand protein-coding genes of *G. potanini* Herzensten, and together with the sequences of four *Gymnocypris* species, all *Gymnocypris* species had close relationship by phylogenetic trees using the ME methods (Figure 1). According to the results based on the mitochondrial DNA cytochrome b gene or mitogenoma sequences, *G. przewalskii* and *G. eckloni* were monophyletic in the trees. Furthermore, *G.dobula* and *G. namensis* also had a close genetic relationship, which were consistent with the findings of previous molecular analyses (Qi et al. 2013; Zhang et al. 2015). Thus, the mitochondrial genome data and phylogenetic analysis of the *G. potanini* Herzensten can enrich the evolutionary research of *Gymnocypris*.

**Figure 1.**
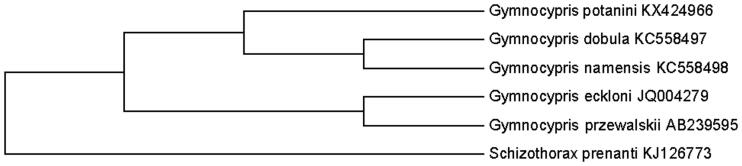
Phylogenetic relationships among five species of Gymnocypris inferred from minimum evolution of deduced amino acid sequences of 12 mitochondrial proteins. The numbers on the branches are bootstrap values for ME.
